# 

**Published:** 1856-07

**Authors:** 


INCLUDING THE

TRANSACTIONS OF THE EPIDEMIOLOGICAL SOCIETY OF LONDON.

EDITED BY

BENJAMIN W. RICHARDSON, M.D.,

PHYSICIAN TO THE ROYAL INFIRMARY FOR DISEASES OF THE CHEST, AND LECTURER ON PUBLIC HYGIENE AT THE GROSVENOR PLACE MEDICAL SCHOOL.

NATIONAL HEALTH IS NATIONAL WEALTH.

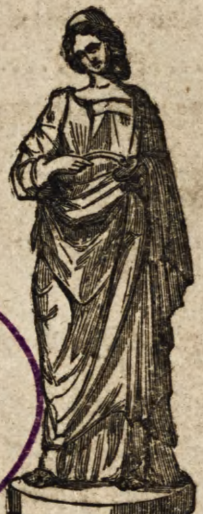

ARMY MEDICAL OC 1 - 1946 LIBRARY

ʽΥΓΙΕΙΑ

*πρεσβίστη μακάρων.*

PRINTED AND PUBLISHED FOR THE PROPRIETOR BY

THOMAS RICHARDS, 37, GREAT QUEEN STREET.

1856.

[PUBLISHED QUARTERLY.—ANNUAL SUBSCRIPTION, 10s., PAYABLE IN ADVANCE.]

